# Effect of Interleukin-15 on CD11b, CD54, and CD62L Expression on Natural Killer Cell and Natural Killer T-Like Cells in Systemic Lupus Erythematosus

**DOI:** 10.1155/2016/9675861

**Published:** 2016-10-26

**Authors:** Syh-Jae Lin, Ji-Yih Chen, Ming-Ling Kuo, Hsiu-Shan Hsiao, Pei-Tzu Lee, Jing-Long Huang

**Affiliations:** ^1^Division of Asthma, Allergy, and Rheumatology, Department of Pediatrics, Chang Gung Children's Hospital, College of Medicine, Chang Gung University, Taoyuan, Taiwan; ^2^Department of Medicine, Division of Allergy, Immunology and Rheumatology, Chang Gung Memorial Hospital, Chang Gung University College of Medicine, Taiwan; ^3^Department of Microbiology and Immunology, Graduate Institute of Biomedical Sciences, College of Medicine, Chang Gung University, Taoyuan, Taiwan; ^4^Division of Allergy, Asthma, and Rheumatology, Department of Pediatrics, Chang Gung Memorial Hospital, Taoyuan, Taiwan; ^5^Chang Gung Immunology Consortium, Chang Gung Memorial Hospital and Chang Gung University, Taoyuan, Taiwan

## Abstract

Adhesion molecules may play an important role in systemic lupus erythematosus (SLE) pathogenesis. We investigated the effect of interleukin- (IL-) 15 on CD11b, CD54, and CD62L expression on natural killer (NK) cells, T cells, and CD56^+^CD3^+^ NKT-like cells from SLE subjects and healthy controls. SLE patients had decreased circulating NK cells and NKT-like cells compared to controls. NK cells from SLE patients showed higher CD11b and CD62L expression compared to controls. IL-15 enhanced CD11b and CD54 but downregulated CD62L expression on NK cells from SLE patients. Similar observations were found for T cells and NKT-like cells. NK cells from SLE patients expressed higher CD56 than controls; both could be further enhanced by IL-15. IL-15 also enhanced CD56 expression of NKT-like cells from SLE patients. A greater degree of IL-15 induced downregulation of CD62L on NKT-like cells noted in SLE patients compared to controls. The percentage of CD11b expressing NK cells and the % inhibition of CD62L expression on NKT-like cells by IL-15 correlated with serum anti-dsDNA levels in SLE patients, respectively. Taken together, we demonstrated the dysfunctional NK and NKT-like cells in SLE patients with regard to CD11b and CD62L expression and their response to IL-15.

## 1. Introduction

Systemic lupus erythematosus (SLE) is characterized by many immunologic abnormalities involving various immune cells like T and B cells [1, 2]. Natural killer (NK) cells, defined by expression of CD56 and lack of CD3, are important effector cells in the innate immune response against infections and tumors [3]. Two subsets of human peripheral blood NK cells have been identified: CD56^dim^ CD16^+^ NK subset is more cytotoxic, while CD56^bright^ subset has the capacity to produce abundant cytokines and plays an important immunoregulatory role [4]. Previous studies have found a decrease in NK cell numbers, impaired NK cytotoxicity, and defects of NK differentiation in SLE patients [5–8].

CD3^+^CD56^+^ NKT-like cells refer to a subset of *αβ* T cells expressing NK activation receptors exhibiting an effector memory phenotype [9, 10]. Like NK cells, NKT-like cells expand in response to viral infection by producing inflammatory cytokines, such as IFN-*γ* [11]. Similar to NK cells, they possess antitumor activity and lyse target cells by secreting perforin and granzyme [12]. NKT-like cells were reported to be decreased in SLE patients. The number of NKT-like cells correlated inversely with SLE disease activity [13].

Interleukin- (IL-) 15 is a pleiotropic common gamma chain signaling cytokine that is important for the activation of CD8^+^ T cells and NK cells [14, 15]. IL-15 plays a* crucial* role in NK differentiation and survival, as in IL-15-deficient mice; the development of NK cells is severely compromised [16]. Patients with SLE have increased serum levels of IL-15, which did not correlate with disease severity [17, 18]. It remains uncertain whether IL-15 may contribute to the pathogenesis of SLE.

Cell adhesion molecules that mediate the leukocyte recruitment to the inflamed tissue and regulate lymphocyte homing may play a pathogenic role in SLE [19, 20]. CD11b is an important integrin that marks NK cell maturation and cytotoxicity [21]. CD54 (ICAM-1) belongs to the immunoglobulin gene superfamily and plays an important role in various inflammatory conditions [22]. CD62L belongs to a member of selectins that is important for NK cells homing to the lymph nodes and also an important marker for NK maturation and response to viral infections [23]. Previous studies have shown that circulating soluble CD54 and CD62L correlated with SLE disease activity [24, 25].

In the present study we examined the expression of CD11b, CD54, and CD62L on NK, T, and NKT-like cells from the peripheral blood of both SLE patients and healthy controls. We sought to determine whether IL-15 would influence the expression of these molecules and their relationship to SLE disease activity.

## 2. Materials and Methods

### 2.1. Study Subjects

Study subjects include 33 SLE patients (*n* = 33) and 17 healthy controls recruited from Chang Gung Memorial Hospital (CGMH), Linkou, Taiwan. The diagnosis of SLE fulfills the 1997 American College of Rheumatology classification criteria [26]. We evaluated the severity of our SLE patients using the systemic lupus erythematosus disease activity index (SLEDAI) scoring method [27]. Laboratory parameters such as C3, C4, and anti-dsDNA were recorded. We obtained heparinized whole blood from each study individual under the preapproval by the institutional research committee at CGMH. Informed consent was provided for all blood donors.

### 2.2. PBMC Incubation

Peripheral blood mononuclear cells (PBMCs) were collected by Ficoll-Hypaque density gradient centrifugation (GE Healthcare, Uppsala, Sweden) within 6 hours of blood drawing. PBMCs were then incubated in RPMI-1640, 10% fetal calf serum in the presence or absence of IL-15 at the concentration of 10 ng/mL (Peprotech, Rocky Hill, USA) for eighteen hours. NK cell viability remains >98% after incubation with IL-15 at 10 ng/mL for 18 hours, while IL-15 at 50 or 100 ng/mL may induce the apoptosis of NK cells (data not shown).

### 2.3. Flow Cytometric Analysis

Following incubation with or without IL-15, PBMCs were harvested, washed, and resuspended for staining. For each experiment, cells were stained with APC-conjugated anti-CD3 antibody (BD Biosciences, San Jose, CA, USA), FITC-conjugated anti-CD56 antibody (BD Biosciences, San Jose, CA, USA), and PE-conjugated anti-CD54, anti-CD11b, and anti-CD62L antibody (Beckman Coulter, Fullerton, CA, USA) for 30 min at 4°C. Cells were then washed twice and analyzed by a Becton Dickinson FACScan analyzer. First, the lymphocyte population was gated to identify CD3-positive and CD3-negative lymphocyte populations. Secondly, the CD3-positive and CD3-negative lymphocyte populations were gated for further analysis of the expression patterns of CD56 and the adhesion molecules. In some experiments when NK cell numbers are adequate, CD56^+^CD3^−^ NK cells were further divided by CD16 and CD56 staining in to 2 groups: CD16^+^CD56^dim^ NK cells (more than 80%) and CD16^dim^ CD56^bright^ NK cells according to the mean fluorescence intensity (MFIs) of CD56 (Figure 3). The % inhibition of CD62L expression by IL-15 was calculated as [MFI of CD62L in medium − MFI of CD62L in the presence of IL-15 (10 ng) for 18 hours/MFI of CD62L in medium] × 100.

### 2.4. Statistics

The Wilcoxon signed rank test was applied for analysis of the responses before and after a treatment, using SPSS 9.0 software. The Mann-Whitney *U* test was used to compare SLE and healthy donor responses. Spearman's rank correlation was applied to detect the association between different parameters. The data are presented as means ± standard error of mean. Data were considered significantly different if *p* was less than 0.05.

## 3. Results

### 3.1. Patient Characteristics and Percentages of NK, T, and NKT-Like Cells

The characteristics of the controls and SLE patients were shown in Table 1. Patients were predominantly female, ages between 12 and 32 years, and had an average disease duration of 8.9 ± 0.7 years. Approximately 57.6% of patients were taking regular corticosteroids and some were receiving methotrexate, azathioprine, or mycophenolate. The percentages of CD56^+^CD3^−^ NK cells from the peripheral blood of SLE patients were lower than those from healthy controls (*p* = 0.008). The percentages of CD56^+^CD3^+^ NKT-like cells from SLE patients were also lower than controls (*p* = 0.038). There was no difference of the percentages of CD56^−^CD3^+^ T cells between SLE patients and controls (*p* = 0.38).

### 3.2. CD11b, CD54, and CD62L Expression on CD56^+^CD3^−^ NK Cells

**Figure 1:**
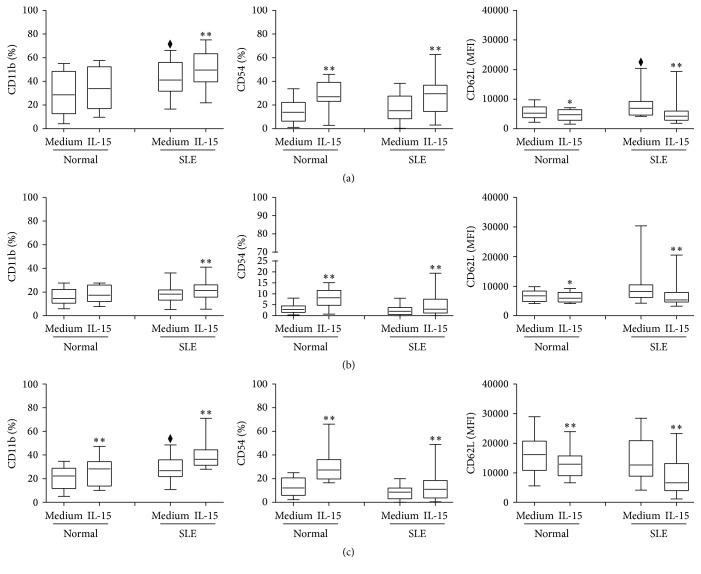
The CD11b, CD54, and CD62L expression on (a) CD56^+^CD3^−^ NK cells, (b) CD56^−^CD3^+^ T cells, and (c) CD56^+^CD3^+^ NKT-like cells from SLE patients and healthy controls. PBMCs were stimulated with IL-15 (10 ng/mL) for 18 hrs. Cells were stained by anti-CD3 and anti-CD56 antibodies and segregated into CD56^+^CD3^−^ NK cells, CD56^−^CD3^+^ T cells, and CD56^+^CD3^+^ NKT-like cells. Surface marker (CD11b, CD54, or CD62L) expression on each cell population was analyzed by flow cytometry. Data was expressed as percent expression (%) ± SEM. ^⧫^ *p* < 0.05 compared to normal; ^*∗*^ *p* < 0.05/^*∗∗*^ *p* < 0.01 compared to medium (normal, *n* = 17; SLE, *n* = 33).

Figure 1(a) shows the CD11b, CD54, and CD62L expression on NK cells from SLE patients and controls. NK cells from SLE expressed higher CD11b compared to controls (42.9 ± 3.1% versus 31.0 ± 5.1%, *p* = 0.032). The expression of CD54 on NK cells from SLE patients was not different from controls (18.2 ± 2.2% versus 14.3 ± 2.5%, *p* = 0.302). As the majority of NK, T, and NKT-like cells expressed CD62L (data not shown), CD62L expression was presented as MFI. The MFI of CD62L on NK cells from SLE patients was higher than controls (9226 ± 1395 versus 5617 ± 658, *p* = 0.033).

IL-15 enhanced the CD11b expression of NK cells from SLE patients (50.1 ± 2.9% versus 42.9 ± 3.1%, *p* = 0.012) but had no effect on CD11b expression of NK cells from controls (34.8 ± 4.9% versus 31.0 ± 5.0%, *p* = 0.055). IL-15 enhances the expression of CD54 on NK cells from both SLE patients (27.4 ± 2.8% versus 18.2 ± 2.2%, *p* < 0.001) and controls (28.2 ± 2.9% versus 14.3 ± 2.5%, *p* = 0.002), respectively. In contrast, IL-15 resulted in a decrease in CD62L expression on NK cells from both SLE patients (5619 ± 1357 versus 9226 ± 1395, *p* = 0.028) and controls (4482 ± 552 versus 5617 ± 658, *p* = 0.001).

### 3.3. CD11b, CD54, and CD62L Expression on CD56^−^CD3^+^ T Cells

CD11b, CD54, and CD62L expression on T cells from SLE patients and controls are shown in Figure 1(b). T cells expressed much lower CD11b and CD54 than did NK cells, both SLE patients and controls alike. T cells from SLE patients expressed comparable CD11b (17.9 ± 1.5% versus 16.1 ± 1.9%, *p* = 0.528), CD54 (2.4 ± 0.4% versus 3.3 ± 0.6%, *p* = 0.151), and CD62L (65.7 ± 3.1% versus 59.0 ± 3.1%, *p* = 0.193) compared to controls. IL-15 enhanced CD54 expression on T cells from SLE patients (4.8 ± 0.9% versus 2.4 ± 0.4%, *p* < 0.001) and controls (8.3 ± 1.0% versus 3.3 ± 0.6%, *p* = 0.001) alike. IL-15 resulted in an increase of CD11b expression (20.7 ± 1.7% versus 17.9 ± 1.5%, *p* = 0.003) and a decrease of CD62L MFI (7205.7 ± 904.7 versus 10767.9 ± 1424.9, *p* < 0.001) on T cells from SLE patients.

### 3.4. CD11b, CD54, and CD62L Expression on CD56^+^CD3^+^ NKT-Like Cells

CD11b, CD54, and CD62L expressions on NKT-like cells from SLE and controls are shown in Figure 1(c). NKT-like cells from SLE patients expressed higher CD11b (28.7 ± 1.9% versus 21.0 ± 2.7%, *p* = 0.043) but comparable CD54 (8.5 ± 1.2% versus 13.2 ± 2.5%, *p* = 0.075) and CD62L (14565 ± 1392 versus 16676 ± 1630 [MFI], *p* = 0.317) compared to controls. Similar to that observed in NK cells, IL-15 enhanced CD11b and CD54 expression on NKT-like cells from both SLE patients (CD11b: 38.4 ± 1.9% versus 28.7 ± 1.9%, *p* < 0.001; CD54: 14.5 ± 2.9% versus 8.5 ± 1.2%, *p* = 0.005) and controls (CD11b: 26.0 ± 3.1% versus 21.0 ± 2.7%, *p* = 0.007; CD54: 30.6 ± 4.2% versus 13.2 ± 2.5%, *p* = 0.007), respectively. IL-15 resulted in a decrease in CD62L MFI on NKT-like cells from both SLE patients (9213 ± 1150 versus 14565 ± 1392, *p* < 0.001) and controls (13151 ± 1214 MFI versus 16676 ± 1630, *p* = 0.002).

### 3.5. IL-15 Enhances CD56 Expression on NK and NKT-Like Cells from SLE

**Figure 2:**
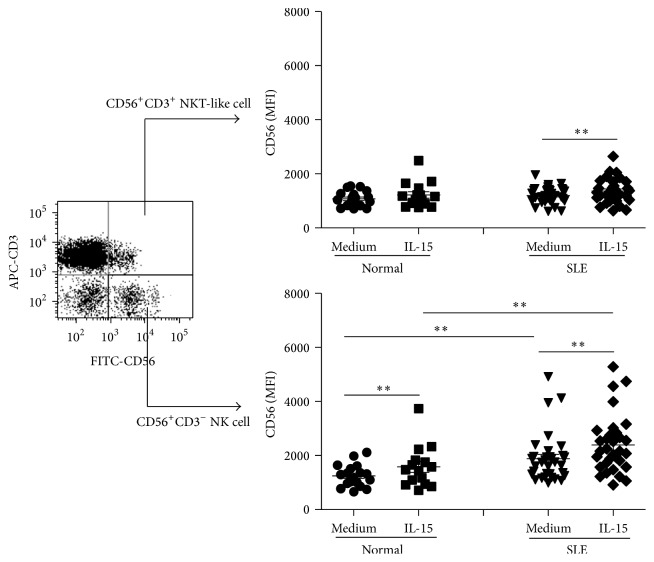
Effect of interleukin- (IL-) 15 on CD56 expression of CD56^+^CD3^−^ NK cells and CD56^+^CD3^+^ NKT-like cells from SLE patients and healthy controls. PBMCs were stimulated with IL-15 (10 ng/mL) for 18 hrs and stained with anti-CD3 and anti-CD56. The CD56^+^CD3^−^ NK and CD56^+^CD3^+^ NKT-like cell population were separated by flow cytometry as illustrated. Data was expressed as mean fluorescence intensity (MFI) ± SEM. ^*∗∗*^ *p* < 0.01 (normal, *n* = 17; SLE, *n* = 33).

We next examine the expression of CD56, another NK marker responsible for adhesive function [28], on NK cells from SLE patients compared to controls. As shown in Figure 2, although NK cells are deficient in numbers compared to controls (Table 1), NK cells from SLE patients exhibited higher CD56 MFI on CD3^−^CD56^+^ NK cells (1886.4 ± 156.7 versus 1250.9 ± 96.8, *p* = 0.002). IL-15 enhanced CD56 MFI on NK cells from both SLE patients and controls. The CD56 MFI on NKT-like cells from SLE patients was comparable to that from controls. IL-15 enhanced CD56 MFI of NKT-like cells from SLE patients (1363 ± 79 versus 1199 ± 52, *p* = 0.001) and had no effect on controls (1218 ± 120 versus 1079 ± 67, *p* = 0.211).

### 3.6. CD56^bright^ NK Cells Were More Responsive to IL-15 Induced Downregulation of CD62L

**Figure 3:**
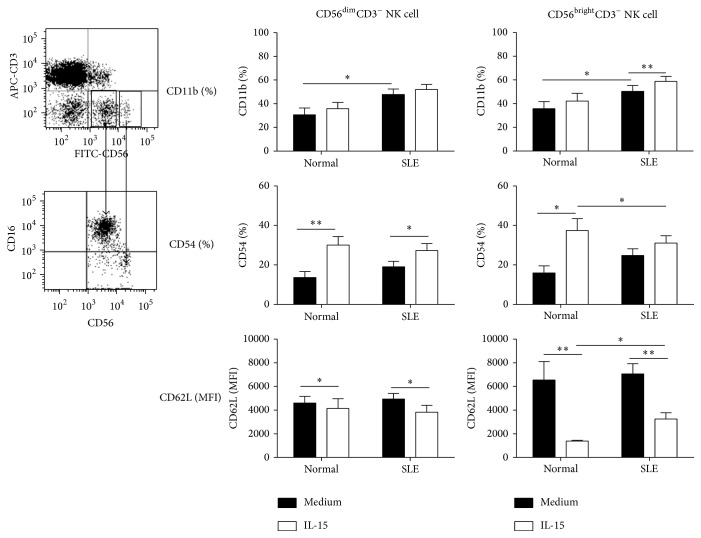
The CD11b, CD54, and CD62L expression on CD56^dim^CD3^−^ and CD56^bright^CD3^−^ NK cells from SLE and healthy donors. PBMCs were stimulated with IL-15 (10 ng/mL) for 18 hrs and stained with anti-CD3 and anti-CD56. A representative profile of how the CD56^dim^ and CD56^bright^ NK populations were gated under flow cytometry is shown. Data was expressed as percent expression (%) ± SEM. ^*∗*^ *p* < 0.05 and ^*∗∗*^ *p* < 0.01 (normal, *n* = 10; SLE, *n* = 17).

When NK cells are further divided into CD56^bright^CD16^−^ and CD56^dim^CD16^+^ subsets (Figure 3), we found that there is no difference of CD11b, CD54, and CD62L expression between CD56^bright^ and CD56^dim^ NK cell subsets in SLE patients and controls alike. For SLE patients, IL-15 enhanced CD11b expression of CD56^bright^ NK subsets (58.8 ± 4.2% versus 50.4 ± 4.9%, *p* = 0.002) but did not affect that of the CD56^dim^ subsets (52.1 ± 4.2% versus 47.8 ± 4.6%, *p* = 0.096). No significant difference of CD54 expression and its response to IL-15 was noted between CD56^bright^ and CD56^dim^ NK subsets. CD56^bright^ NK cells expressed greater CD62L than did CD56^dim^ subsets. IL-15 downregulates CD62L expression on CD56^bright^ NK subsets to a greater extent than on that of on CD56^dim^ subsets from SLE patients (28.3 ± 4.7% versus 20.6 ± 3.4%, *p* = 0.023).

### 3.7. Greater CD62L Downregulation by IL-15 in NKT-Like Cells from SLE Patients

**Figure 4:**
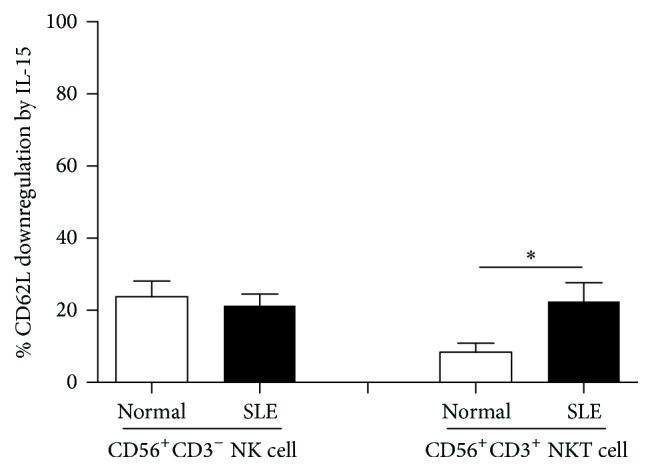
The inhibition of CD62L expression by IL-15 on CD56^+^CD3^−^ NK cells and CD56^+^CD3^+^ NKT-like cells from SLE and healthy donors. PBMCs were stained with anti-CD3, anti-CD56, and anti-CD62L antibodies and were analyzed under flow cytometry. % CD62L downregulation by IL-15 is calculated as [MFI of CD62L in medium − MFI of CD62L in the presence of IL-15 (10 ng/mL) for 18 hours/MFI of CD62L in medium] × 100. ^*∗*^ *p* < 0.05 (normal, *n* = 10; SLE, *n* = 17).

We next compare the degree of CD62L inhibition by IL-15 of NK cells and NKT-like cells (Figure 4). The % downregulation of CD62L MFI by IL-15 in NK cells was comparable between SLE patients and controls (21.3 ± 3.3% versus 23.8 ± 4.4%, *p* = 0.66). However, NKT-like cells from SLE were more susceptible to IL-15 induced downregulation compared to controls (22.4 ± 5.2% versus 8.4 ± 2.5%, *p* = 0.018).

### 3.8. Correlation of CD11b and CD62L Expression on NK and NKT Cells and Disease Activity

**Figure 5:**
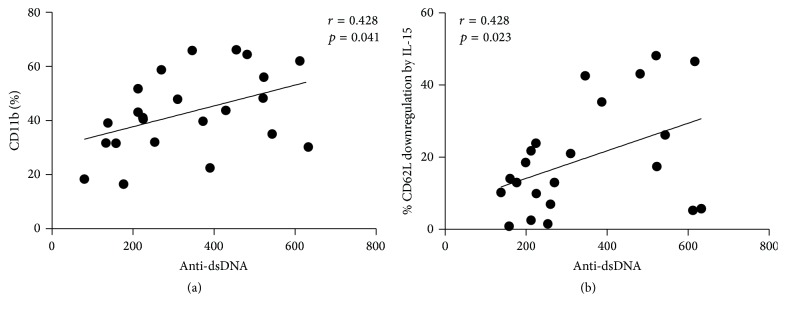
Correlation between (a) serum anti-dsDNA and the percentages of CD11b^+^ NK cells and (b) serum anti-dsDNA and the % inhibition of CD62L MFI on NKT-like cells by IL-15 calculated as [MFI of CD62L in medium − MFI of CD62L in the presence of IL-15 (10 ng/mL) for 18 hours/MFI of CD62L in medium] × 100.

To evaluate the clinical relevance of adhesion molecule expression on NK and NKT-like cells in SLE patients, we investigate relationships of the percentages of NK and NKT-like cell bearing CD11b and CD62L and SLE-related laboratory parameters by regression analysis. As shown in Figure 5, univariate analysis showed that the percentages of CD11b^+^ NK cells correlated with serum anti-dsDNA levels, in SLE patients (*r* = 0.428, *p* = 0.041), while the % inhibition of CD62L MFI by IL-15 of NKT-like cells also correlates with serum anti-ds DNA levels (*r* = 0.374, *p* = 0.043).

## 4. Discussion

In the present study, we compared the expression of adhesion molecules CD11b, CD54, and CD62L on NK, T, and NKT-like cells from SLE patients and healthy controls. We also determined the effect of IL-15, an immunoregulatory cytokine, on adhesion molecule expression. IL-15 may directly affect immune cells or indirectly by upregulating other inflammatory mediators. We have shown that IL-15 could enhance tumor necrosis factor-alpha and interferon-gamma production of NK cells [29]. We have also demonstrated that IL-15 induced interferon-gamma but not IL-4 production from human iNKT cells [30].

In agreement with the previous studies [31, 32], we found that the percentages of NK cells and NKT-like cells are decreased in PBMC from SLE patients compared to controls. However, The percentages of T cells were comparable to controls. CD11b, but not CD54 and CD62L expression on NK cells from SLE patients, was increased compared to controls. NK cells progress from an immature CD27^+^CD11b^−^ stage to an intermediate CD27^+^CD11b^+^ stage and finally to a CD27^−^CD11b^+^ stage [33]. CD11b^+^ NK cells are more readily responsive to cytokine-mediated activation during viral infection [34]. Our findings suggest that CD11b^+^ bearing NK cells may play a pathogenic role in SLE.

T cells from SLE have been shown to have aberrant signaling, abnormal cytokine secretion [35]; we found however that CD11b, CD54, and CD62L expression of T cells from SLE was comparable to controls. CD3^+^CD56^+^ NKT-like cells have been reported to be increased in certain autoimmune conditions like Behçet's diseases [36, 37]. Similar to that observed with NK cells, we found that CD3^+^CD56^+^ NKT-like cells from SLE patients exhibited higher CD11b expression compared to the corresponding controls.

Consistent with our previous work [38, 39], IL-15 enhanced CD54 expression of NK, T, and NKT-like cells from SLE patients and controls alike, suggesting its ability to promote cell migration and cytotoxicity. We found that IL-15 enhanced CD11b expression of NK cells and T cells from SLE patients, respectively, an effect not observed with healthy controls. Contrary to that observed in CD11b and CD54, we found that IL-15 downregulates CD62L expression on NKT cells and NKT-like cells, from SLE patients and controls.

Although the percentages of NK cells decreased, we found that the CD56 MFI was elevated on SLE NK cells compared to controls, consistent with Schepis et al. [7]. We further demonstrated that CD56 expression on NK cells from SLE patients could be further enhanced with IL-15 stimulation. IL-15 also preferentially enhanced MFI of CD56 of NKT-like cells from SLE patients. Thus, IL-15 may play a disease-promoting role by enhancing CD56 expression on NK and NKT-like cells of SLE patients.

Previous studies have shown the dysregulation of CD56^bright^ NK cells in SLE patients [7, 31]. We found that CD56^bright^ NK cells from SLE patients expressed higher CD11b and CD62L than the CD56^dim^ counterparts. CD11b on CD56^bright^ NK cells from SLE patients but not controls could be enhanced by IL-15. CD56^bright^ NK cells were also more readily susceptible to IL-15 compared to their CD56^dim^ counterparts with regard to CD62L downregulation.

There was no difference in the % inhibition of CD62L by IL-15 on NK cells between SLE patients and controls. However, a greater degree of IL-15 induced downregulation of CD62L on NKT-like cells was noted in SLE patients compared to controls. IL-15 resulted in shedding of CD62L on NK cells and especially NKT-like cells to the circulation which may aggravate the tissue inflammation in SLE. Serum soluble CD62L has been reported to be an SLE disease marker [40, 41].

Correlation analysis revealed that CD11b expression on NK cells correlated with serum anti-dsDNA levels in SLE patients. The % inhibition of CD62L MFI by IL-15 of NKT-like cells also correlated with serum anti-dsDNA levels. IL-15 may aggravate SLE disease severity by promoting CD62L shedding from the surface of NKT-like cells.

Cytokine inhibition may be used as a strategy for treating SLE. Ma et al. demonstrated a beneficial effect of anti-IL-15 in the treatment of murine lupus [42]. Humax-IL15, a human IgG1 anti-IL-15 monoclonal antibody, has been shown to improve symptoms in patients with rheumatoid arthritis [43]. Our finding suggests that IL-15 may be a potential target for immunotherapy against SLE.

Taken together, we demonstrated the dysfunctional NK and NKT-like cells in SLE patients with regard to CD11b and CD62L expressions and their response to IL-15. IL-15 may aggravate inflammation by preferentially upregulating CD11b and CD56 and downregulating CD62L on NK and NKT-like cells in SLE patients. Antagonist to IL-15 may provide a therapeutic option to ameliorate the progression of SLE.

## Figures and Tables

**Table 1:** Characteristics of controls and patients with SLE.

Characteristics	Controls (*n* = 17)	SLE patients (*n* = 33)
Sex (male/female)	0/17	0/33
Age, mean (range)	29.3 ± 0.9 (27–34)	22.5 ± 0.9 (12–32)
SLEDAI score, median (range)	NA	7 (0~25)
C3, median (range)	NA	66.7 (21–106)
C4, median (range)	NA	9.7 (2.1–32.9)
Anti-dsDNA, median (range)	NA	309.9 (79.6–632.8)
Average disease duration	NA	8.9 ± 0.7
Taking regular corticosteroids	NA	57.6%
Cell population		
CD56^+^CD3^−^ NK cells (%)^#^	7.3 ± 0.9%	4.4 ± 0.6%^*∗∗*^
CD56^−^CD3^+^ T cells (%)	71.8 ± 1.4%	67.2 ± 2.8%
CD56^+^CD3^+^ NKT-like cells (%)	3.5 ± 0.6%	2.2 ± 0.3%^*∗*^

^*∗*^ *p* < 0.05 and ^*∗∗*^ *p* < 0.01 compared to controls; ^#^the percentages of NK cells, T cells, and NKT-like cells in total lymphocytes.
